# *Akkermansia muciniphila* for the Prevention of Type 2 Diabetes and Obesity: A Meta-Analysis of Animal Studies

**DOI:** 10.3390/nu16203440

**Published:** 2024-10-11

**Authors:** Ethan Liu, Xiangming Ji, Kequan Zhou

**Affiliations:** 1 Department of Nutrition and Food Science, Wayne State University, Detroit, MI 48202, USA; ethanliu0307@gmail.com; 2 Department of Nutritional Sciences, The College of Health and Human Development, The Pennsylvania State University, University Park, PA 16802, USA

**Keywords:** probiotic, *Akkermansia*, obesity, type 2 diabetes, animals, meta-analysis

## Abstract

Background: More than half of the states in the U.S. report that over 30% of adults are obese. Obesity increases the risk of many chronic diseases, including type 2 diabetes, hypertension, and cardiovascular disease, and can even reduce one’s lifespan. Similarly, the prevalence of type 2 diabetes follows a comparable trend. As a result, researchers are striving to find solutions to reduce obesity rates, with a particular focus on gut health, which has been previously linked to both obesity and type 2 diabetes. Recent studies suggest that *Akkermansia muciniphila* (Akk) may have a positive probiotic effect on preventing the onset of type 2 diabetes and obesity. Methods: We conducted a quantitative meta-analysis of 15 qualified animal studies investigating the effects of Akk administration as a probiotic. Results: The statistical analyses showed that Akk administration significantly reduced body weight gain by 10.4% and fasting blood glucose by 21.2%, while also significantly improving glucose tolerance by 22.1% and increasing blood insulin levels by 26.9%. However, our analysis revealed substantial heterogeneity between the control and experimental groups across all subgroups. Conclusions: Overall, Akk appears to be effective at reducing the onset of type 2 diabetes and diet-induced obesity. Long-term studies with larger sample sizes are needed to confirm these beneficial effects, as the current animal studies were of short duration (less than 20 weeks).

## 1. Introduction

The alarming surge in obesity and diabetes rates within the USA is reaching critical levels, with 41.9% and 14.7%, respectively among American adults [1,2]. In 2022, 43% of adults in the world were overweight and 16% were living with obesity [3]. Moreover, obesity stands as a significant precursor to diabetes, amplifying the urgency of this health crisis [3]. Both conditions demand sustained attention and comprehensive care, presenting formidable challenges to public health. Addressing this pressing issue necessitates the development of innovative, sustainable long-term strategies. There has been strong evidence suggesting that both obesity and diabetes have been strongly linked to gut microbiota, the vast community of microbes in our intestines [4]. Studies have shown that lean mice gained significant weight after transferring gut bacteria from obese mice, suggesting that gut microbiota play a key role in managing body weight [5,6]. Other studies in animals and humans demonstrate an inverse relationship between obesity and diversity of gut microbiota, specifically symbionts including genera Bifidobacteria, Lactobacillus, and Verrucomicrobiota (in which Akk belongs to) [7,8]. In addition, gut microbiota has been shown to alter low-grade inflammation and insulin resistance upon modification via dietary interventions [9]. In particular, a group of gut bacterial metabolites such as short-chain fatty acids (SCFAs) are believed play a role in mediating the effects of gut microbiota [10]. For instance, diabetes patients were found to have a reduced population of SCFAs-producing gut bacteria, suggesting a potential link of gut bacteria with impaired blood sugar control [4]. Common SCFAs include acetate, butyrate, and propionate, and their anti-obesity and anti-diabetes bioactivities have been widely studied. These findings also suggest that a potential strategy to manage obesity and type 2 diabetes is targeting gut microbiota.

Akk is a gram-negative gut bacterium belonging to the phylum Verrucomicrobia. It is one of the most abundant gut bacteria in humans, representing 3–5% of the microbial community. A. muciniphila was first isolated and identified in 2004 in purified mucin as an oval-shaped, strictly anaerobic bacterium. Akk has the ability to utilize mucus glycans as carbon sources to grow. As a mucin-degrading bacterium, Akk in turn stimulates the growth of the protective gut lining and enhances gut barrier functions [11,12]. Akk has recently gained increased research interest due to its potential benefits against obesity and metabolic syndrome. Research has found that Akk is more abundant in healthy subjects than in diabetic and obese patients. Further support comes from intervention studies that have also found an inverse correlation between Akk abundance and body weight, metabolic syndrome, and type 2 diabetes [13,14,15,16].

**Figure 1:**
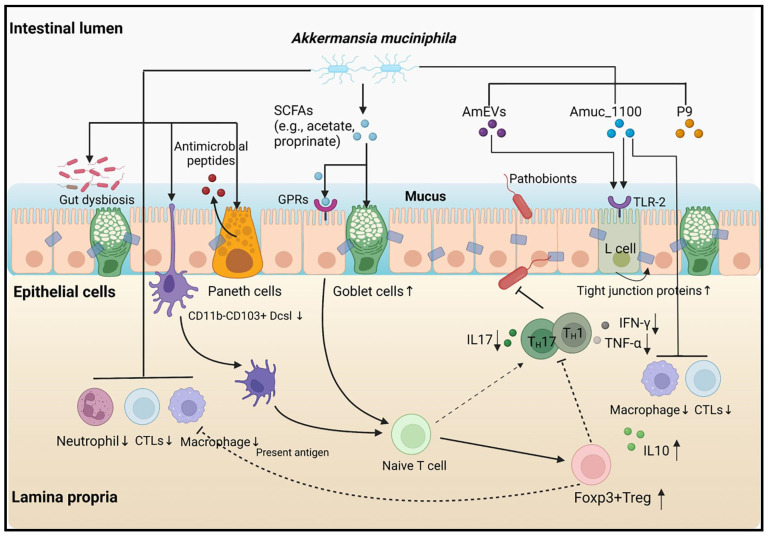
Various pathways and models associated with *A. muciniphila* [7].

Despite the growing body of evidence suggesting that Akk is a promising probiotic, the exact mechanisms by which it exerts its beneficial impact on health have not been fully elucidated (Figure 1). Studies have shown that Akk promotes the gut production of short-chain fatty acids (SCFAs), improving insulin resistance and host metabolism—factors linked to obesity and diabetes [12,13,14,15]. Moreover, Akk has been shown to modify the gut microbiota by stimulating other beneficial gut bacteria such as Bifidobacteria, thereby promoting a healthier gut ecosystem [13].

There have been a growing number of studies investigating the beneficial role of Akk on obesity and diabetes [7]. Animal studies have been particularly encouraging. For instance, dietary Akk supplementation has been shown to reduce body weight gain, fasting blood glucose, and insulin resistance in mice fed with a high-fat diet [14,15]. Similar findings have been demonstrated in other studies, suggesting the protective role of Akk against obesity and diabetes, though the exact mechanisms are not clear. A recent human trial, while demonstrating improved insulin sensitivity and reduced cholesterol in overweight/obese participants, did not show significant changes in weight or body fat composition [16]. This discrepancy highlights the need for a more comprehensive analysis of Akk effects on obesity and diabetes. A previous review that compared the effects of Akk on various metabolic diseases found a positive association with the onset of type 2 diabetes following Akk supplementation. However, this association lacked strong evidence [17]. To address this, our meta-analysis aims to systematically evaluate the latest research and compare the effects of Akk administration in different animal studies. We will employ the most comprehensive subgroup analyses possible to provide a more definitive understanding of the impact of Akk.

## 2. Materials and Methods

### 2.1. Literature Search Strategy and Inclusion Criteria

This meta-analysis investigated the effect of Akk dietary intake on obesity and type 2 diabetes, focusing on animal studies that generated quantitative data. This study was conducted strictly following the guidelines of Preferred Reporting Items for Systematic Reviews and Meta-Analyses (PRISMA) [18]. The databases PubMed, EMBASE, Web of Science, and Cochrane Library were searched for animal trials on Akk to prevent obesity and type 2 diabetes.

Publications from the inception of the databases until February 2024 were collected. We employed a combination of terms from medical subject headings (MeSH) and keywords. The search strategy encompassed subject headings “Obesity” or “diabetes mellitus” or “metabolic syndromes” in conjunction with identifiers such as “animals” or “mice” or “rats.” The search keywords included “probiotic(s)” and “*Akkermansia muciniphila*”. All retrieved articles were systematically compiled using Endnote software (20th version). Additionally, we searched bibliographies to ensure inclusivity of relevant literature not initially captured by the database search.

Inclusion criteria: (1) randomized controlled trials in mice; (2) studies employing the Akk probiotic in experimental groups, with a placebo control group for comparison; (3) alternatively, studies utilizing a substrate containing Akk; and (4) studies reporting outcomes related to the risk and development of diabetes or obesity. Exclusion criteria: (1) duplicate studies; (2) studies that were non-blinded trials; (3) studies where the probiotics composition of the intervention was not specified. Article screening and data extraction were completed by 2 reviewers using Covidence software (https://www.covidence.org (accessed on 10 March 2024)), as recommended by the Cochrane Handbook for Systematic Reviews of Interventions and others [19].

### 2.2. Data Extraction and Quality Assessment

Two researchers independently selected the studies, resolving any disagreements through consultation with a third researcher. Data from the selected studies were extracted into a shared spreadsheet by one reviewer and validated by a second reviewer. The data extraction utilized a standardized form by an independent researcher. The primary outcome was the incidence or biomarkers of obesity or type 2 diabetes, along with any metabolic syndrome factors, upon administration of Akk in animal models. The secondary outcome included the incidence of adverse events or outlier outcomes. Additional extracted data encompassed demographics, indications for antibiotics, probiotic species and dosage, and duration of probiotic administration. Data were extracted as continuous variables [20].

The quality of the selected studies was assessed using the Cochrane Handbook for Systematic Reviews of Interventions [21]. Previously used study quality scales were adapted to evaluate study quality and possible bias risk [22,23]. The methodological assessment scale and its elements were informed by guidelines and publications from rigorous biomedical research such as the STAIR and RIGOR guidelines [24,25]. Taking into account the distinctive nature of the selected animal studies on Akk, the following parameters were considered for study quality assessment: presence or absence of exclusion statement, study pre-registration, inclusion of both sexes, aged and/or compromised animals, sample size, control of diet in the experimental group, clear description of infusion parameters (e.g., rate and/or duration, and dosage), and conflict of interest statements.

### 2.3. Statistical Analyses

Data analyses were performed using RStudio (version 4.3.3, Ann Arbor, MI, USA), with plots generated using the robumeta, metafor, and dplyr packages. Due to the variability in probiotic durations and infusion parameters, a random-effect meta-analysis model utilizing the Derismonian-Laird estimator was selected for all endpoints. The standardized mean differences (SMDs) were used for calculating effect sizes, with Hedge’s G applied to correct for bias in studies with small sample sizes. Pooled relative risk (RR) and the 95% confidence interval (CI) were calculated using a random-effects model (DerSimonian-Laird method [26]) or a fixed-effects model (Mantel-Haenszel method [27]).

Meta-regression was conducted to evaluate heterogeneity, taking into account variables such as probiotic duration, infusion parameters (including temperature and rate volume), and methodological quality. Sensitivity and subgroup analyses were carried out in cases where unexpected or extreme outcomes were identified in the forest plots. Heterogeneity among the included studies was assessed using the Chi-squared (X^2^) test and the I^2^ statistic [28,29]. A *p* < 0.1 or I^2^ > 50% indicated substantial heterogeneity, warranting the adoption of a random-effects model. In the absence of substantial heterogeneity, a fixed-effects model was applied. Publication bias was evaluated through funnel plots and further evaluated using the Begg and Egger tests [22,23].

## 3. Results

### 3.1. Eligible Studies

**Figure 2:**
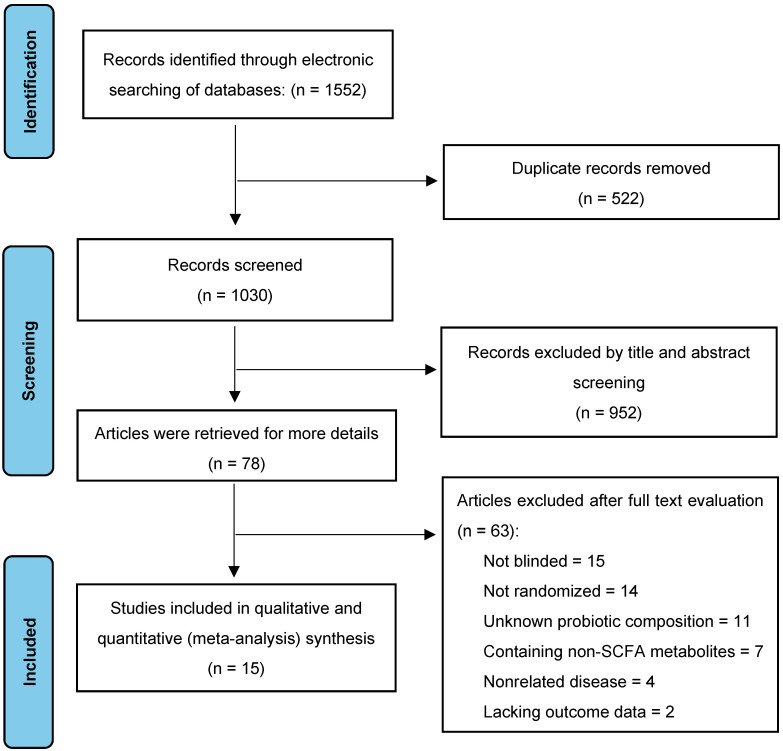
Selection of studies for the meta-analysis.

A comprehensive literature search was conducted in January 2024, yielding 1552 citations across four databases: PubMed (461), Cochrane Library (52), EMBASE (336), and Web of Science (703). After removing duplicates and conducting title and abstract screenings, 1030 studies were deemed potentially relevant for further evaluation. Of these, 952 of these studies were excluded due to irrelevance or methodological shortcomings. A full-text review of the remaining 78 studies led to the exclusion of 63 studies due to methodological issues (15 non-blinded, 14 non-randomized, 11 unknown probiotic composition) or irrelevant outcomes (seven reporting non-SCFA metabolites, four involving unrelated diseases, and two with missing outcome data). Ultimately, 15 studies met our inclusion criteria, encompassing a total of 586 animals and were selected for data analysis and extraction [30,31,32,33,34,35,36,37,38,39,40,41,42,43,44]. The search flow details are depicted in Figure 2.

### 3.2. Study Characteristics

Table 1 summarizes the characteristics of the selected individual studies, such as animal models, sample size, and Akk treatment conditions (e.g., prior antibiotic use, single treatment or combination with other supplements, dosage, and duration). Regarding animal models used for Akk intervention, C57BL/6J was the most prevalent (73%), followed by db/db, NOD mice, and other less common models (27%). Notably, one study utilized a streptozotocin-induced diabetic model in Sprague–Dawley rats for oral Akk administration. Methodologically, 83% of the studies reported randomization, 60% mentioned exclusions, 47% disclosed conflicts of interest, 26% ensured complete blinding (during Akk administration and outcome assessment), and 11% included the mortality rate for both experimental and placebo groups (Table 1). None of the studies performed a priori group size calculations.

**Figure 3:**
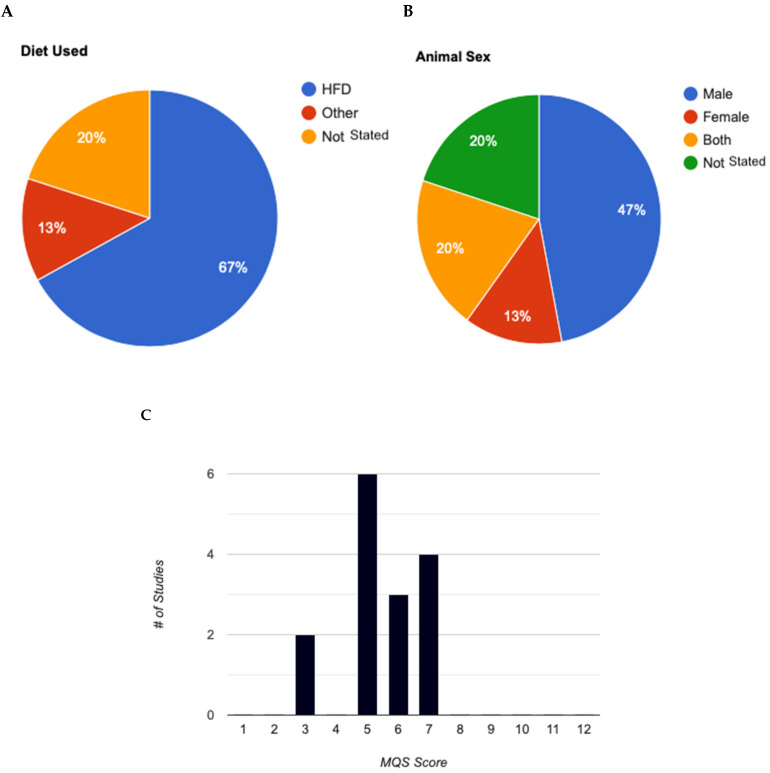
Analysis of experimental characteristics used in animal models for *Akkermansia muciniphila* studies. (**A**): Breakdown of diets used in animal studies. (**B**): Animal sexes used in mouse animal studies. (**C**): Evaluation of methodological quality score for animal studies (max score = 12). MQS, Methodological Quality Score.

The experimental groups were predominantly fed a high-fat diet (HFD), comprising over 60% fat compared to carbohydrates (Figure 3A). Most studies employed young, male C57BL/6J, except for two studies which included females but did not consider biological sex as a variable parameter or specify the number of females per group (Figure 3B).

All studies measured changes in body weight during Akk intervention. Body composition (lean and fat mass) was assessed in 84% of the studies, highlighting its significance as an outcome measure for Akk intervention. Interestingly, four studies employed antibiotics prior to probiotic administration, likely aimed to create a more favorable intestinal environment for robust Akk colonization. The characteristics of interest for the enrolled studies were ranked from most to least prevalent to provide an overview of the current literature characteristics of selected studies (Table 2).

### 3.3. Quality Assessment

The methodological quality of these studies was assessed using the STAIR and RIGOR guidelines, specifically designed to evaluate the rigor and robustness of preclinical research, thereby ensuring reliable translation of findings into human studies [28,29]. Of the 15 eligible studies, only 10 employed blinding to minimize detection bias. Reporting on other potential biases varied among the studies. Unfortunately, none of the included studies explicitly mentioned blinding procedures. For outcomes like obesity, which can be visually apparent, it is unclear what specific tools or methods were used to ensure blinding. Future studies should incorporate more rigorous blinding procedures, such as using blinded assessors for subjective outcomes and objective measurement tools for outcomes like obesity, to minimize the risk of bias and enhance the quality of the evidence.

Higher risk of attrition bias and other concerns were noted in studies that: 1. lacked clear methods for handling missing outcome data and outliers (three out of 15 studies); 2. had a short follow-up period (one out of 15 studies); 3. experienced excessive or unbalanced loss of subjects during the study (six out of 15 studies); 4. utilized a small sample size (five out of 15 studies); and 5. had unbalanced baseline characteristics between groups (four out of 15 studies). Nine studies assessed glucose tolerance, a key indicator of the body’s ability to regulate blood sugar levels. Additionally, 13 studies measured serum insulin levels, and nine studies assessed fasting blood glucose levels.

### 3.4. Analysis of Methodological Quality

Table 1 and Table 2 provide a detailed overview of the methodological quality scale and the analysis of studies incorporating experimental elements. None of the included studies explicitly mentioned the specific randomization and concealment methods used. Future studies should explicitly report randomization and concealment methods to enhance the study quality. Table 3 presents the methodological quality scores (MQS) of the selected studies. The overall median score was 5, indicating low to moderate methodological quality across the studies. Scores ranged from three to seven (out of a maximum of 12 points) as illustrated in Figure 3C. Characteristics of C75BL/6J Mice Model Studies in Akk. All studies, except one, administered Akk immediately after initiating a high-fat diet (HFD) containing 60% of energy from fat. The one exception did not use a HFD but instead administered an antibiotic prior to Akk administration. For studies that included additional supplements, such as metformin or tocotrienol, Akk was administered concurrently with the supplements. In these cases, the mice received either an HFD with 800 mg of tocotrienol or an HFD with 200 mg of metformin, along with Akk.

### 3.5. Impact of Akk on Body Weight

Ten studies examined changes in body weight during and after Akk administration. One study used rats, while the other nine studies used mice as the animal model. The study on rats showed that Akk supplementation significantly reduced body weight by an average of 25%, likely owing to a greater average body weight in rat models than mice models. In the nine mice studies, an average weight reduction of 3.8 g was observed (Table 4). The total number of mice in the control groups across all studies was 135, compared to 155 in the treatment groups. The average body weight in the Akk groups was 32.2 g, while the average weight of the controls was 36.4 g, suggesting a reduction of average body weight gain by 10.4%.

Normalized effect sizes varied across studies, with most studies showing positive effect sizes, suggesting that the treatment generally had a beneficial effect. However, it is important to note that five out of the nine studies reported non-significant changes in overall body weight compared to baseline. This inconsistency suggests significant variability across the studies, with body weight changes ranging from a 0.08 g increase to an 11.0 g decrease following Akk treatment.

**Figure 4:**
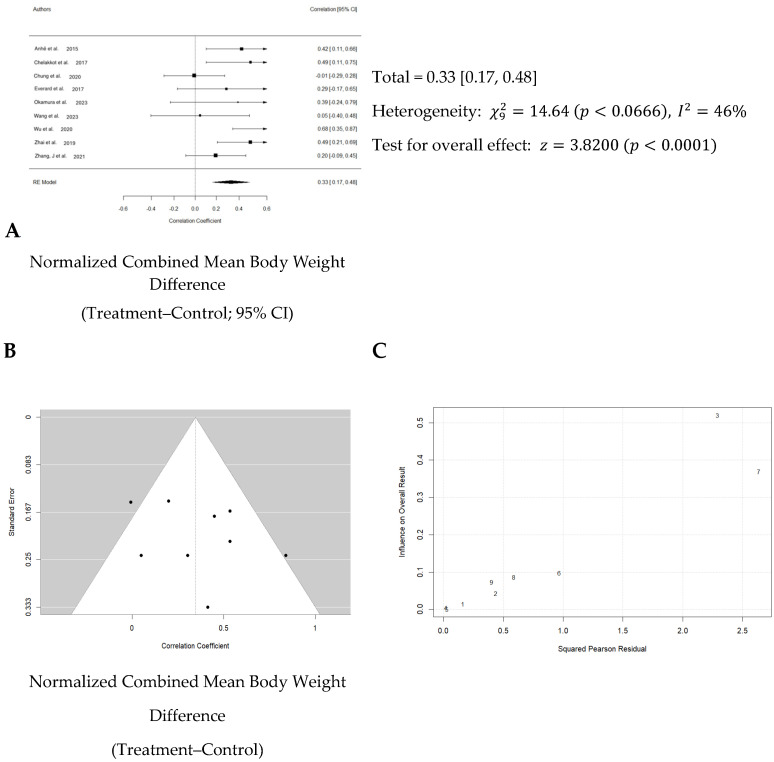
Quantitative analysis of studies that assessed body weight using a random-effects meta-analysis. (**A**): Forest plot of studies investigating body weight effects (normalized mean difference ± 95% CI) [31,32,33,34,36,40,41,42,43]. Effect size estimates were heterogenous, likely owing to study design differences. (**B**): Funnel plot used to assess publication bias. These results suggest that the studies used in the meta-analysis had relatively low publication bias (i.e., the symmetric distribution of closed circles). (**C**): Baujat plot used to assess the studies that contributed most to heterogeneity. These results suggest that studies Chung et al. 2020 [33] and Wu et al. 2020 [41] likely had moderating variables that contributed the most to the heterogeneity.

Overall, the weighted mean effect size of 0.33 (95% CI: 0.17–0.48) indicates a statistically significant reduction in body weight gain due to Akk treatment. A forest plot analysis indicated a significant reduction in body weight gain among mice treated with Akk compared to controls (*p* < 0.001, Figure 4A). However, substantial heterogeneity existed among studies (I^2^ = 46%), and the funnel plot suggested low publication bias (Figure 4B). The Baujat plot identified the studies by Chung et al. and Wu et al. as primary contributors to this heterogeneity (Figure 4C).

### 3.6. Impact of Akk on Glucose Tolerance

Nine studies evaluated the effect of Akk on glucose tolerance (Table 5). In total, 111 animals were in the control groups, while 123 received treatment. On average, Akk treatment led to a 22.1% improvement in glucose tolerance, as estimated by the 2 h area under the curve (AUC) for blood glucose following a glucose tolerance test. Since the exact AUC values were not provided in all the selected studies, these estimates were derived from the individual study graphs using an image analysis program (https://imagej.nih.gov/ij/, accessed on 10 March 2024). The effect sizes varied. While three studies showed significant reductions in AUC, the majority reported only minor, non-significant decreases.

**Figure 5:**
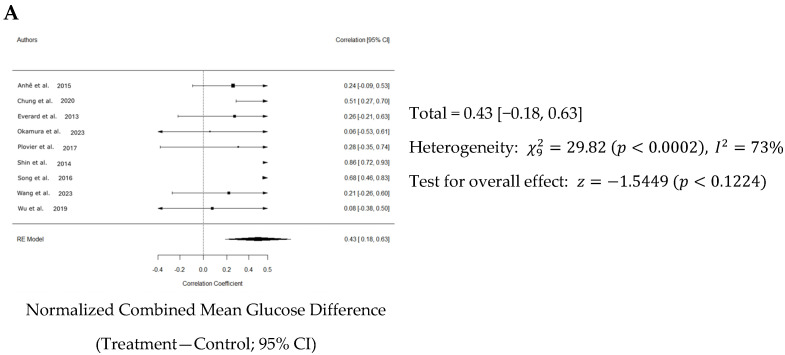

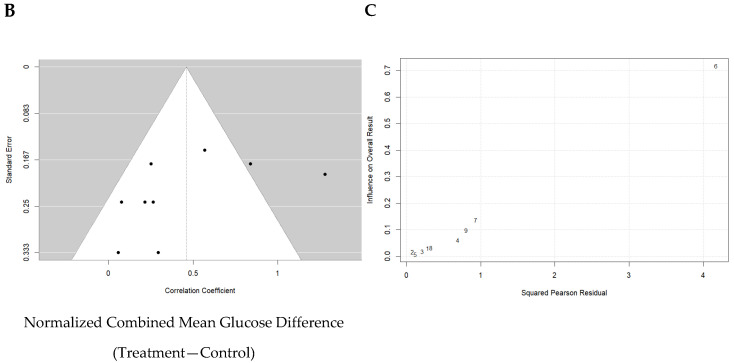
Quantitative analysis of studies that assessed glucose tolerance using a random-effects meta-analysis. (**A**): Forest plot of studies investigating glucose tolerance (normalized mean difference ±95% CI) [31,33,34,36,37,38,39,40,41]. Effect size estimates were heterogenous, likely owing to study design differences. (**B**): Funnel plot used to assess publication bias. These results suggest that the studies used in the meta-analysis had relatively low publication bias. (**C**): Baujat plot used to assess the studies that contributed most to heterogeneity. These results suggest that the study Shin et al. 2014 [38] likely had moderating variables that contributed the most to the heterogeneity.

Overall, a weighted mean effect size of 0.347 (95% CI: 0.18–0.63) indicated a statistically significant reduction in blood glucose levels due to Akk treatment. Forest plot analysis confirmed this, revealing a pooled effect size of 0.43 (95% CI: 0.18–0.63; *p* < 0.001, Figure 5A). A random-effects model was used to account for heterogeneity between studies, which was substantial (I^2^ = 73%). Nonetheless, the funnel plot suggested low publication bias (Figure 5B). The study by Shin et al. primarily contributed to the observed heterogeneity (Figure 5C).

### 3.7. Impact of Akk on Blood Insulin

Thirteen studies examined circulating insulin hormone levels. As presented in Table 6, the total number of animals in the control groups across all studies was 168, compared to 200 in the treatment groups. The data suggest that Akk treatment stimulated blood insulin production in the animals by 26.9%, as shown by the higher mean level of 2.6 µIU/mL in the treatment groups compared to 1.9 µIU/mL in the control groups.

**Figure 6:**
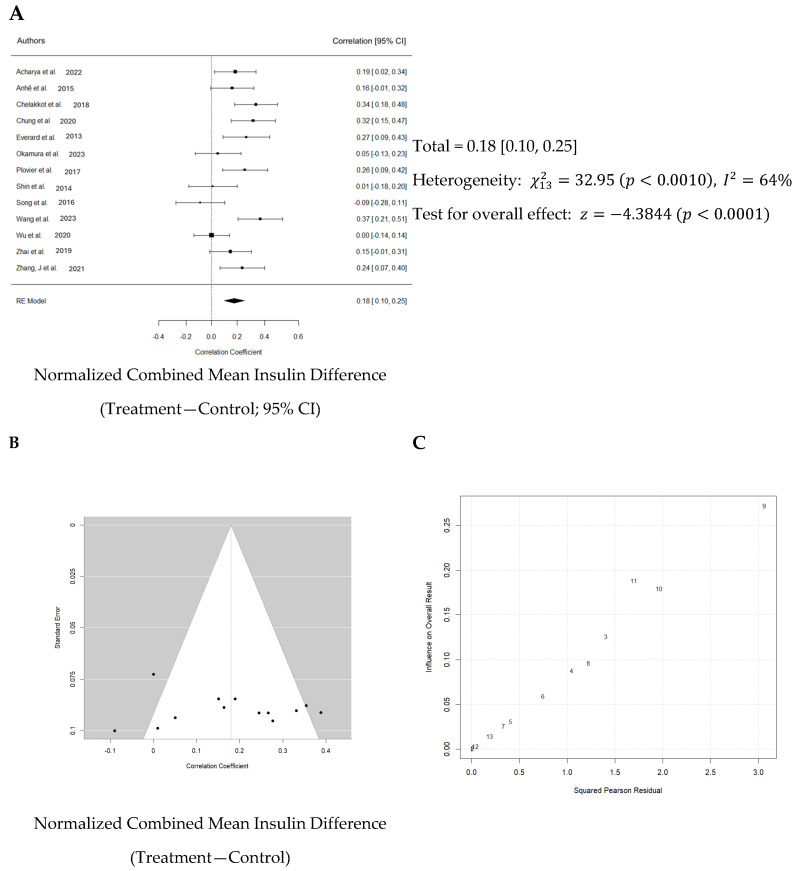
Quantitative analysis of studies that assessed insulin hormone levels using a random-effects meta-analysis. (**A**): Forest plot of studies investigating insulin levels (normalized mean difference ±95% CI) [30,31,32,33,34,36,37,38,39,40,41,42,43]. Effect size estimates were very heterogenous, likely owing to study design differences and differences in probiotic duration. (**B**): Funnel plot used to assess publication bias. These results suggest that the studies used in the meta-analysis had relatively low publication bias. (**C**): Baujat plot used to assess the studies that contributed most to heterogeneity. These results suggest that studies Shin et al. 2014 [38], Song et al. 2016 [39], and Wang et al. 2023 [40] likely had moderating variables that contributed the most to the heterogeneity.

Normalized effect sizes varied across studies, with most studies showing positive effect sizes, suggesting that the treatment generally had a beneficial effect. Nevertheless, only seven of 13 studies showed that the effect was significant. The weighted mean effect size of 0.827 with a confidence interval of 0.10 to 0.25 indicates a statistically significant reduction in blood glucose levels due to Akk treatment. The forest plot also revealed a significant increase in blood insulin levels in animals administered Akk compared to controls (*p* < 0.001) as shown in Figure 6A. The analysis showed a pooled effect size of 0.18 (95% confidence interval: 0.10–0.25). There was significant variation in the study results (I^2^ = 64%). Additionally, the analysis suggests a potential publication bias (Figure 6B). These results indicate that the studies by Shin et al., Song et al. and Wang et al. contributed the most to the observed heterogeneity (Figure 6C). 

### 3.8. Impact of Akk on Fasting Blood Glucose

Nine studies investigated the impact of Akk on fasting blood glucose levels. Detailed information on these assessment methods can be found in Table 7. The analysis revealed that Akk treatment significantly reduced fasting blood glucose levels by 21.2%, as shown by the lower mean level of 114.7 mg/dL in the treatment groups compared to 145.5 mg/dL in the control groups.

**Figure 7:**
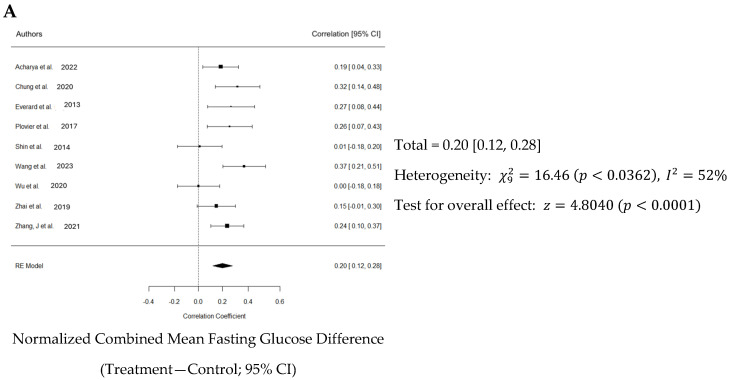

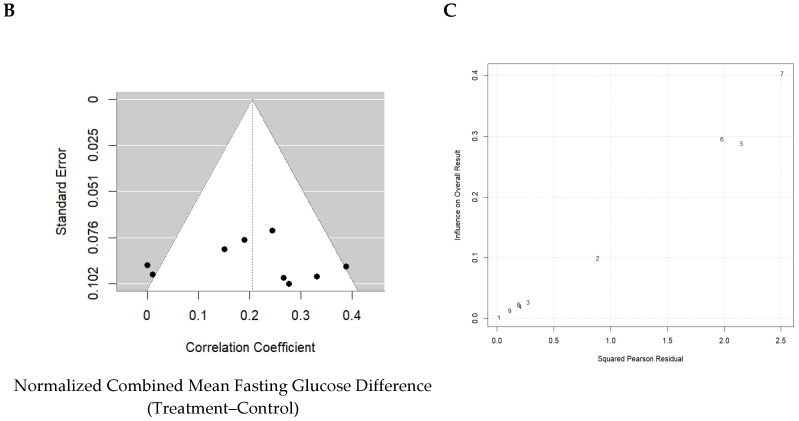
Quantitative analysis of studies that assessed fasting blood glucose using a random-effects meta-analysis. (**A**): Forest plot of studies investigating fasting blood glucose (normalized mean difference ± 95% CI) [30,33,34,37,38,40,41,42,43]. Effect size estimates were heterogenous, likely owing to study design differences. (**B**): Funnel plot used to assess publication bias. These results suggest that the studies used in the meta-analysis had relatively low publication bias. (**C**): Baujat plot used to assess the studies that contributed most to heterogeneity. These results suggest that studies Shin et al. 2014 [38], Wang et al. 2023 [40], and Wu et al. 2020 [41] likely had moderating variables that contributed the most to the heterogeneity.

Normalized effect sizes varied across studies, with most studies showing positive effect sizes, suggesting that the treatment generally had a beneficial effect. Seven of nine studies showed that the effect was significant. The weighted mean effect size of 0.623 with a confidence interval of 0.12 to 0.28 indicates a statistically significant reduction in blood glucose levels due to Akk treatment. The forest plot also revealed a significant increase in blood insulin levels in animals administered Akk compared to controls (*p* < 0.001) as shown in Figure 7A. The analysis showed a pooled effect size of 0.20 (95% confidence interval: 0.12–0.28). There was significant variation in the study results (I^2^ = 52%). However, the analysis suggests a low risk of publication bias, indicating a fair representation of both positive and negative findings (Figure 7B). Three studies by Shin et al., Wang et al. and Wu et al. contributed the most to the observed heterogeneity (Figure 7C).

## 4. Discussion

This meta-analysis underscores the multifaceted benefits of Akk intake in mice subjected to a high-fat diet. Overall, dietary supplementation with Akk, as analyzed from nine selected studies, significantly reduced body weight by an average of 10.4%. The dosage of Akk ranged from 2 × 10^8^ to 5 × 10^10^ CFU/day, which aligns with the dosages commonly used in most probiotic animal studies. Most of the selected studies on Akk used dosages of 2 × 10^8^ and 1 × 10^10^ CFU. However, two studies by Wang et al. [40] and Zhang et al. [43] utilized a higher dose of 5 × 10^10^ CFU in mice. Interestingly, both studies showed that Akk had no effect on body weight, despite other studies with lower doses showing a beneficial effect in reducing body weight gain. These results suggest that there is no dose-dependent relationship of Akk in the prevention of body weight gain. The median duration of Akk treatment across the selected nine animal studies was 14 weeks, ranging from 6 to 20 weeks. The most prominent effects of Akk were observed during treatment durations of 6 to 12 weeks. The results suggest that Akk exerts its effect in reducing body weight gain during the early stages of obesity development (as early as 6 weeks), with the probiotic effect likely diminishing over time. For instance, Zhang’s study [43] extended Akk treatment to 20 weeks at higher dose, but showed no inhibiting effect on body weight gain. Obesity is a chronic disease that requires a long-term solution. The current animal studies were all of short duration (less than 20 weeks). Long-term studies are needed to verify Akk’s effect on obesity development.

The effect of Akk supplementation on the prevention of type 2 diabetes is reflected in its significant reduction of fasting blood glucose (by 21.2%) and improvement in glucose tolerance (by 22.1%), suggesting that Akk treatment is effective in preventing type 2 diabetes. Nine studies have measured fasting blood glucose. Except for two studies, all others showed a significant reduction in fasting blood glucose with Akk treatment. These two studies had a short treatment duration of 6 weeks, while the others were longer, suggesting that Akk may require more than 6 weeks to demonstrate a significant effect on blood glucose. No dose–response relationship was observed across the tested doses between 2 × 10^8^ and 5 × 10^10^ CFU/day in the nine studies. It was unclear if and how treatment duration affected the efficiency of the probiotic during administration. Additionally, a discrepancy emerged regarding the inclusion of antibiotics, which was the predominant method. In the study by Acharya et al., the administration of the antibiotic before the probiotic was actually more effective than administering the probiotic by itself [30]. Few authors provided clear justifications for the model choice and dosing parameters utilized in their studies.

Nine studies measured glucose tolerance. The meta-analysis showed that Akk significantly improved glucose tolerance in mice by reducing the 2 h AUC by 22.1%. However, there was substantial variation in the results across studies, as reflected by I^2^ = 73%. Indeed, six of nine studies showed modest but non-significant effects. The median duration of Akk treatment across studies was 12 weeks, ranging from 6 to 16 weeks. Shin et al. found that six weeks of Akk treatment significantly reduced DIO mice 2 h AUC by an estimated 27.3%. This mouse study of Akk treatment showed the most prominent effect among the studies with a normalized effect size of 0.856. Plovier et al. also investigated six weeks of Akk treatment on mouse glucose tolerance but with a lower dose (2 × 10^8^ CFU/d versus 1 × 10^10^ CFU/d in Shin’s study) and found no significant effect. The other two studies with significant effects by Akk treatment also used doses at or above 1 × 10^10^ CFU/d, suggesting that the Akk treatment dose may need to be no less than 1 × 10^10^ CFU/d to be effective. It should be noted that one study by Wang et al. used a model of FFAR4 knockout mice, which has been shown to cause severely impaired glucose tolerance under high-fat feeding conditions. FFAR4 regulates glucagon-like peptide 1 secretion by modifying Akk abundance. However, our meta-analysis showed that Akk treatment at 5 × 10^10^ CFU/d for 12 weeks in Wang’s study did not significantly improve glucose tolerance. There was substantial heterogeneity among the studies. The studies differed in terms of doses, duration, sample size, and other factors, which could significantly influence the results. Further research is needed to confirm the consistency and magnitude of the effect of Akk on glucose tolerance. Overall, fasting blood glucose decreased by 21.2%, whilst blood insulin increased by 26.9% when comparing control to treatment groups likely because insulin and blood glucose are inversely correlated.

Thirteen studies investigated the effect of Akk treatment on blood insulin levels in animals. The meta-analysis revealed a significant increase in blood insulin levels, from 1.9 to 2.6 µg/mL. Notably, three studies—Zhang et al. [43], Chung et al. [33], and Zhai et al. [42]—demonstrated strong treatment effects, with effect sizes of 1.64, 1.44, and 1.17, respectively. Interestingly, all three studies also reported significant reductions in fasting blood glucose levels. Additionally, some studies explored the anti-inflammatory efficacy of Akk; however, due to the heterogeneity of inflammation biomarkers across blood and tissue, a meta-analysis on the anti-inflammatory effects was not feasible.

One of the primary objectives of this meta-analysis was to identify the variables that contribute to the efficacy of Akk, such as changes in body weight and treatment duration. Surprisingly, the random-effects meta-regression model is not able to determine these expected variables. A random-effects model was selected for meta-regression because of the aforementioned variables in study design, such as probiotic duration, infusion parameters (including temperature, rate volume), and overall methodological quality. Notably, treatment duration which has been a critical determinant of efficacy in previous studies (both preclinical and clinical), did not emerge as a significant factor in our analysis [20,28]. This discrepancy might be due to the limited exploration of treatment durations in the analyzed studies. Only one study specifically investigated the impact of different treatment durations, reporting no significant effect of the probiotic beyond 14 weeks [18].

There are several limitations leading to inclusive results in our meta-regression models. First, the limited number of studies assessing fasting blood glucose levels restricted our ability to conduct a comprehensive meta-regression due to constraints in degrees of freedom [45]. Second, the presence of substantial variability among studies can compromise the power of meta-regression. Our analysis revealed significant variation between studies, as indicated by heterogeneity statistics in our primary endpoints model. This could be due to factors such as system errors among different laboratories, such as experiment duration, sample preparation, and administration parameters of Akk. Third, the animal models used (age, sex, and species) were inconsistent. This significant methodological and model variability among the studies made it challenging to identify clear cause-and-effect relationships. Fourth, most studies employed young, male C57BL/6J mice. Only two studies included female mice, but neither considered biological sex as a variable parameter or specified the number of females per group. Consequently, the potential effects of Akk on female animals remain unclear. Moreover, the inconclusive results regarding microbiota suggest that Akk’s influence on gut microbiota might be more critical than its direct metabolic effects on parameters such as blood glucose levels. On the other hand, the current data make it difficult to definitively determine the key factors influencing treatment outcomes.

## 5. Conclusions

Given the increasing prevalence of metabolic morbidities such as type 2 diabetes and diet-induced obesity, this meta-analysis aimed to evaluate the potential of Akk as a novel therapeutic intervention in animal models. Our findings strongly suggest that Akk is a promising candidate for mitigating these metabolic diseases, demonstrating efficacy in preventing body weight gain and type 2 diabetes. To further elucidate the clinical implications of these findings, future studies should incorporate a broader range of experimental parameters. Factors such as age, sex, and treatment duration may influence treatment outcomes [27,45]. Additionally, comprehensive, long-term outcome assessments are essential for a more complete understanding of Akk’s effects on body weight and type 2 diabetes. Therefore, we recommend conducting large-scale animal model validation studies to determine the reliability and generalizability of our meta-analysis findings. These future studies should address the limitations identified in the current literature and contribute to a more comprehensive understanding of the clinical potential of Akk.

## Figures and Tables

**Table 1:** Characteristics of the enrolled studies.

References	Risk of Bias	Setting	Sample Size (Treatment/Placebo Group)	Obesity/Type 2 Diabetes Definition	High-Fat Diet Composition %	Antibiotic(s)	Time from Antibiotic/HFD to Probiotic, d	Probiotic Species	Dosage per Day	Probiotic Duration	Experimental Period
Acharya et al. [30]	Low	C57BL/6J mice, female, 10-week-old	7/14	WHO	60%	Various	14	*A. muciniphila*	2 × 10^8^ CFU	10 d	4 wk
Anhê et al. [31]	Low	C57BL/6 mice, male	18/18	WHO	65%	None	2	*A. muciniphila* (Cranberry extract)	1.5 × 10^10^ CFU	2 wk	8 wk
Chelakkot et al. [32]	Low	C57BL/6 mice, male, 6–8-week-old	12/12	Other definition	60%	None	14	*A. muciniphila*	4 × 10^10^ CFU	2 wk	12 wk
Chung et al. [33]	Low	C57BL/6 mice, male	24/24	WHO	58%	None	N/A	*A. muciniphila* (metaformin, tocotrienol)	2 × 10^10^ CFU, 8 × 10^10^ CFU	12 wk	14 wk
Everard et al. [34]	Low	C57BL/6 mice, male	10/10	WHO	60%	None	N/A	*A. muciniphila*	2 × 10^8^ CFU	4 wk	14 wk
Hänninen et al. [35]	Medium	NOD/MrkTac mice, 7–8-week-old	16/21	WHO	N/A	None	N/A	*A. muciniphila*	1 × 10^10^ CFU	2 wk	18 wk
Okamura et al. [36]	Medium	7-week-old db/db mice	6/6	WHO	N/A	None	N/A	*A. muciniphila* (milk)	1 × 10^10^ CFU	8 wk	16 wk
Plovier et al. [37]	Medium	mice, male	6/6	WHO	HFD	None	N/A	*A. muciniphila*	2 × 10^8^ CFU	2 wk	6 wk
Shin et al. [38]	Low	C57BL/6J mice	15/15	Other definition	HFD	None	N/A	*A. muciniphila* (metaformin)	1 × 10^10^ CFU	6 wk	6 wk
Song et al. [39]	Low	C57BL/6J mice	12/24	WHO	HFD	None	N/A	*A. muciniphila*	1 × 10^10^ CFU	4 wk	14 wk
Wang et al. [40]	Low	Villin Cre and Gko, adult male mice	10/10	WHO	N/A	None	N/A	*A. muciniphila*	5 × 10^10^ CFU	6 wk	12 wk
Wu et al. [41]	Medium	C57BL/6J	10/10	WHO	HFD	None	N/A	*A. muciniphila*	1 × 10^10^ CFU	2 wk	6 wk
Zhai et al. [42]	Low	C57BL/6 7-week-old male mice	10/30	WHO	N/A	None	N/A	*A. muciniphila*	2 × 10^10^ CFU	9 d	14 wk
Zhang, J et al. [43]	Low	C57BL/6J 8-week-old male mice	28/21	WHO	HFD	None	N/A	*A. muciniphila*	5 × 10^10^ CFU	15 wk	20 wk
Zhang, L et al. [44]	Medium	Sprague-Dawley rats	105/77	Other definition	66%	Streptozocin	3	*A. muciniphila*	1 × 10^10^ CFU	4 wk	8 wk

**Table 2:** Methodological quality scale reporting elements for *Akkermansia muciniphila* research in type 2 diabetes and obesity, and percentage adherence.

Element	% Adherence
Control of diet	100
Blinding to outcome(s)	87
Complete reporting of infusion parameters	87
Group randomization	80
Includes type 2 diabetes	67
Exclusion statement	60
Conflict of interest statement	47
Use of male and female animals	40
Blinding to type 2 diabetes	40
Includes obesity	33
Blinding to obesity	23
Use of aged animals	0
Sample size calculation	0
A priori study registration	0
Comorbidities	0

**Table 3:** Statistical summary comparison and total percentage of reported MQS elements (✓ = yes, χ = no).

References	Pre-Registration	Exclusion Statement	Both Sexes	Old Age	Sample Size Calcuation	Group Randomization	Comorbidities	Outcome(s) Blinding	Disease Blinding	Control of Diet	Infusion Parameters	Conflict of Interest	Total Score (Max. 12)
Acharya et al. [30]	χ	χ	χ	χ	χ	✓	χ	✓	✓	✓	✓	✓	6
Anhê et al. [31]	χ	✓	χ	χ	χ	✓	χ	✓	✓	✓	✓	✓	7
Chelakkot et al. [32]	χ	χ	χ	χ	χ	✓	χ	χ	✓	✓	✓	✓	5
Chung et al. [33]	χ	✓	χ	χ	χ	✓	χ	✓	χ	✓	✓	✓	5
Everard et al. [34]	χ	✓	χ	χ	χ	✓	χ	✓	χ	✓	✓	✓	5
Hänninen et al. [35]	χ	χ	✓	χ	χ	χ	χ	✓	✓	✓	✓	χ	5
Okamura et al. [36]	χ	✓	✓	χ	χ	✓	χ	✓	✓	✓	✓	χ	7
Plovier et al. [37]	χ	χ	χ	χ	χ	χ	χ	✓	χ	✓	✓	χ	3
Shin et al. [38]	χ	✓	✓	χ	χ	✓	χ	✓	χ	✓	✓	χ	6
Song et al. [39]	χ	χ	✓	χ	χ	✓	χ	✓	χ	✓	χ	✓	5
Wang et al. [40]	χ	✓	χ	χ	χ	✓	χ	✓	✓	✓	✓	✓	7
Wu et al. [41]	χ	✓	✓	χ	χ	✓	χ	✓	✓	✓	✓	χ	7
Zhai et al. [42]	χ	✓	χ	χ	χ	✓	χ	✓	✓	✓	✓	χ	6
Zhang, J et al. [43]	χ	✓	χ	χ	χ	✓	χ	✓	✓	✓	✓	χ	5
Zhang, L et al. [44]	χ	χ	✓	χ	χ	χ	χ	χ	✓	✓	χ	χ	3
Total (%)	0	60	40	0	0	80	0	87	67	100	87	47	

**Table 4:** Descriptive characteristics of studies that assessed body weight.

References	Duration (wk)	Control (n)	Experimental (n)	Control Body Weight (g)	Treatment Body Weight (g)	Normalized Effect Size	95% Confidence Interval
Anhê et al. [31]	8	18	18	27.8	23.5	0.421	0.11, 0.66
Chelakkot et al. [32]	12	12	12	45	40	0.489	0.11, 0.75
Chung et al. [33]	14	24	24	38.44	38.52	−0.008	−0.29, 0.28
Everard et al. [34]	14	10	10	24	21	0.294	−0.17, 0.65
Okamura et al. [36]	16	6	6	48	44	0.391	−0.24, 0.79
Wang et al. [40]	12	10	10	27	26.5	0.049	−0.40, 0.48
Wu et al. [41]	6	10	10	39.5	32.5	0.685	0.35, 0.87
Zhai et al. [42]	14	10	30	55	50	0.489	0.21, 0.69
Zhang, J et al. [43]	20	28	21	31.5	29.5	0.196	−0.09, 0.45
Summary statistic	Median = 13	Σ = 135	Σ = 155	Mean = 36.4	Mean = 32.2	XWeighted = 0.331	XWeighted = 0.17, 0.48

**Table 5:** Descriptive characteristics of studies that assessed glucose tolerance.

References	Duration (wk)	Control (n)	Experimental (n)	Control Blood Glucose (AUC mg/dL) (120 min)	Treatment Blood Glucose (AUC mg/dL) (120 min)	Normalized Effect Size	95% Confidence Interval
Anhê et al. [31]	8	18	18	10,500	6200	0.245	−0.09, 0.53
Chung et al. [33]	14	24	24	57,000	48,000	0.513	0.27, 0.70
Everard et al. [34]	14	10	10	34,500	30,000	0.257	−0.21, 0.63
Okamura et al. [36]	16	6	6	11,000	10,000	0.057	−0.53, 0.61
Plovier et al. [37]	6	6	6	40,000	35,000	0.285	−0.35, 0.74
Shin et al. [38]	6	15	15	55,000	40,000	0.856	0.72, 0.93
Song et al. [39]	14	12	24	24,000	12,000	0.685	0.46, 0.83
Wang et al. [40]	12	10	10	12,200	8500	0.211	−0.26, 0.60
Wu et al. [41]	6	10	10	11,860	10,530	0.076	−0.38, 0.50
Summary statistic	Median = 12	Σ = 111	Σ = 123	Mean = 28,500	Mean = 22,200	XWeighted = 0.347	XWeighted = 0.18, 0.63

**Table 6:** Descriptive characteristics of studies that assessed blood insulin.

References	Duration (wk)	Control (n)	Experimental (n)	Treatment Insulin Levels (µg/mL)	Control Insulin Levels (µg/mL)	Normalized Effect Size	95% Confidence Interval
Acharya et al. [30]	4	7	14	2.5	2.1	0.587	0.02, 0.34
Anhê et al. [31]	8	18	18	0.8	0.6	0.134	−0.01, 0.32
Chelakkot et al. [32]	12	12	12	2.7	2	0.703	0.18, 0.48
Chung et al. [33]	14	24	24	4	2.5	1.406	0.15, 0.47
Everard et al. [34]	14	10	10	2.2	1.9	0.234	0.09, 0.43
Okamura et al. [36]	16	6	6	2.5	2.2	0.469	−0.13, 0.23
Plovier et al. [37]	6	6	6	1.9	1.5	0.469	0.09, 0.42
Shin et al. [38]	6	15	15	2.7	1.9	0.938	−0.18, 0.20
Song et al. [39]	14	12	24	2.8	2	0.938	−0.28, 0.11
Wang et al. [40]	12	10	10	2.9	2.1	0.938	0.21, 0.51
Wu et al. [41]	6	10	10	3	2.2	0.938	−0.14, 0.14
Zhai et al. [42]	14	10	30	3.1	2.1	1.172	−0.01, 0.31
Zhang, J et al. [43]	20	28	21	3.2	1.8	1.641	0.07, 0.40
Summary statistic	Median = 12	Σ = 168	Σ = 200	Mean = 2.6	Mean = 1.9	XWeighted = 0.827	XWeighted = 0.10, 0.25

**Table 7:** Descriptive characteristics of studies that assessed fasting blood glucose levels.

References	Duration (wk)	Control (n)	Experimental (n)	Control Fasting Blood Glucose (mg/dL)	Treatment Fasting blood Glucose (mg/dL)	Normalized Effect Size	95% Confidence Interval
Acharya et al. [30]	4	7	14	178	156	0.502	0.04, 0.33
Chung et al. [33]	14	24	24	100	58	0.958	0.14, 0.48
Everard et al. [34]	14	10	10	175	135	0.912	0.08, 0.44
Plovier et al. [37]	6	6	6	260	160	2.281	0.07, 0.43
Shin et al. [38]	6	15	15	122	112	0.228	−0.18, 0.20
Wang et al. [40]	12	10	10	124	114	0.228	0.21, 0.51
Wu et al. [41]	6	10	10	125	115	0.228	−0.18, 0.18
Zhai et al. [42]	14	10	30	126	116	0.228	0.01, 0.30
Zhang, J et al. [43]	20	28	21	99.5	66.5	0.753	0.10, 0.37
Summary statistic	Median = 12	Σ = 120	Σ = 140	Mean = 145.5	Mean = 114.7	XWeighted = 0.623	XWeighted = 0.12, 0.28

## Data Availability

The original contributions presented in this study are included in the article, further inquiries can be directed to the corresponding author.
